# Risk behaviors and sports facilities do not explain socioeconomic differences in childhood obesity: a cross-sectional study

**DOI:** 10.1186/1471-2458-14-1181

**Published:** 2014-11-19

**Authors:** Romana Albaladejo, Rosa Villanueva, Lourdes Navalpotro, Paloma Ortega, Paloma Astasio, Enrique Regidor

**Affiliations:** Department of Preventive Medicine and Public Health, Faculty of Medicine, Universidad Complutense de Madrid, Ciudad Universitaria, 28040 Madrid, Spain; Health’s Sciences, Universidad Antonio de Nebrija, Madrid, Spain; CIBER Epidemiología y Salud Pública (CIBERESP), Madrid, Spain

**Keywords:** Socioeconomic context, Obesity risk behaviors, Sports facilities, Childhood overweight, Childhood obesity

## Abstract

**Background:**

To assess whether the relationship between neighborhood socioeconomic context of residence and childhood obesity is explained by family socioeconomic position, risk behaviors and availability of sports facilities.

**Methods:**

Based on the income and educational level of residents in the neighborhoods of the city of Madrid, two indicators of socioeconomic context were calculated using the information about income and education and grouped into quartiles. In a sample of 727 children aged 6–15 years, the relationship of these indicators with overweight and obesity was studied using multilevel logit models.

**Results:**

With respect to children and adolescents living in neighborhoods having higher per capita incomes or higher population percentages with university education those living in neighborhoods having lower per capita incomes or lower population percentages with university education had age- and sex-adjusted odds ratios (ORs) of overweight that were 1.84 (95% CI, 1.03-3.29) and 1.68 (0.95-2.94) times higher, respectively. After adjustment for family socioeconomic position, unhealthy diet and physical inactivity, these ORs fell to 1.80 (0.99-3.29) and 1.56 (0.87-2.79), respectively. In the case of obesity, the age- and sex-adjusted ORs in these quartiles of both indicators of socioeconomic context were 3.35 (1.06-10.60) and 3.29 (1.03-10.52), respectively, rising to 3.77 (1.12-12.70) and 3.42 (1.00-11.68) after adjustment for the remaining variables. The highest OR was observed in the third quartile, except in the case of the relationship between per capita income and obesity. No relationship between the number of sport facilities per 1,000 population and physical inactivity was observed.

**Conclusion:**

The socioeconomic context is associated with obesity but not with overweight children in Madrid. The relationship is not explained by family socioeconomic position, risk behaviors and availability of sports facilities.

## Background

Due to its high prevalence, childhood obesity is an important public health problem in many developed countries [1]. The relevance of this is because obesity in children increases their risk of obesity in adulthood [2] and development of chronic diseases [3, 4]. Among the factors that increase the likelihood of obesity in childhood population are the social determinants of health. One of the social determinants of health most closely studied in the last decade is the socioeconomic context of the area of residence. Different studies on adolescents and youth have highlighted the fact that areas with a lower income level display the highest prevalences of obesity and/or overweight, regardless of family socioeconomic position [2, 5–8]. In general, this association is maintained after adjusting for family socioeconomic position [9–11], a finding which suggests that some characteristics of a given area of residence may be implicated in the development of obesity.

Among the environmental characteristics of area of residence that have been identified as possible obesogens are those related with diet and physical activity [4, 12–16]. The availability of supermarkets and stores selling healthy foods has been reported to be lower in areas with lower income than in higher income areas [17], with the result that the price of such foods is correspondingly higher [4]. It has likewise been shown that there are fewer sports facilities in lower- than in higher-income areas, so that the probability of engaging in physical activity is also lower [17].

While most of the above studies were conducted in the USA and United Kingdom [7, 9], although recent studies have begun to appear from other European countries [4, 18–21]. In one of these studies, conducted in Spain, it was observed that the geographic variation in the availability of sports facilities could contribute to the explanation of the relationship between socio-economic context of the area of residence and obesity in children and adolescents [11]. Yet, the unit of analysis used in that study was province, which has a median population size of around 500,000 inhabitants, and this level of aggregation may not be the most suitable for studying whether proximity to infrastructures that promote healthy behaviors in a given area of residence might account for the association found. Accordingly, this study set out to study the children and adolescents of the city of Madrid, with the aim of investigating the relationship between socioeconomic context of neighborhood of residence and overweight/obesity, and assessing whether this possible relationship is explained by family socioeconomic position, obesity-related risk behaviors, and availability of sports facilities.

## Methods

In this study, we used the population interviewed in the 2005 City of Madrid Health Survey. This is an observational study. The Ethical Committee is not necessary. The information is anonymous.

Here individuals aged under 16 years were selected by two-stage cluster sampling, with stratification by census districts; these constituted the first-stage units. The census districts were selected with a probability proportional to their population size, while respondents within each district were chosen by simple random selection. The interviews were conducted at the homes of the persons selected. Questionnaires were completed by one of the parents or, where this was not possible, by the person's guardian. For the purpose of our study, we selected a total of 727 children aged 6–15 years distributed across 119 neighborhoods in the city of Madrid.

In the above survey, every boy and girl was weighed and measured, and their body mass index (BMI) was then calculated on the basis of these data. Overweight and obesity were respectively defined using the international BMI cut-off points established for children and youth [22]. Based on data collected in the survey, we used two measures of family socioeconomic position, namely, the educational level and occupation of the primary family earner. According the first measure children were classified as having 2nd-cycle secondary and postsecondary and university education, or less than 2nd-cycle secondary education. And according the second measure children were classified as being non-manual workers (managers, businessmen, university-qualified professionals, self-employed persons, administration personnel and supervisors) or skilled, semi-skilled and unskilled manual workers.

Physical inactivity and watching TV have been shown to be risk factors for childhood obesity. In the questionnaire, respondents were asked, "Which of these possibilities best describes the frequency with which the child does some physical activity or sport in his/her free time?" The four possible replies were: "1. No exercise"; "2. He/she does some physical or sports activity less than once per month"; "3. He/she does some physical or sports activity one or more times per month"; and, "4. He/she does some physical or sports activity one or more times per week". These responses were grouped into the following two categories: no physical activity (first reply); or, some activity (any of the other alternatives). This measure of physical activity has previously been used and has shown a pattern by age, sex and socioeconomic status similar to that observed in studies of other countries in which they have used a different instrument of measurement [23]. In the survey, interviewees were also asked how many hours per day the children spent watching television. Based on the answers, children were allocated to one of two categories, i.e., those who watched television for two hours or less per day, and those who watched television for more than two hours per day.

The frequency of weekly intake of different foods was also recorded. Based on fruit and vegetable intake, participants were grouped according to whether such food was consumed less than three times per week or, three or more times per week. Similarly, respondents were asked about the type of breakfast usually eaten. Children were grouped into those who had no breakfast or had only milk, and those who, apart from milk, had a complete breakfast including fruit, juice or some other food in addition to milk and toast or cookies.

Two indicators were obtained which reflected the socioeconomic context of the Madrid neighborhoods. These were per capita income as indicator of wealth and the percentage of the population with university education as indicator of human capital. Per capita income was estimated by the Madrid Regional Institute of Statistics based on tax records for the year 2000 [24]. Neighborhoods were grouped into quartiles according to per capita income. The percentage of the population with university education was estimated on the basis of the 2001 population census. Neighborhoods were also grouped into quartiles according to this percentage. Each respondent was then assigned to a quartile of each indicator, based on his/her neighborhood of residence.

The number of sports facilities per 1,000 population was estimated for each neighborhood. Based on the quartiles of the distribution of this rate, a categorical variable was then calculated, with each subject being assigned to a quartile according to his/her neighborhood of residence. Information on the number of sports facilities in each neighborhood was obtained from the most recent National Census of Sports Facilities, undertaken in 2005 [25].

We first assessed the relationship between the indicators of socioeconomic context and overweight and obesity. And then we assessed the relationship between the indicators of socioeconomic context and different characteristics of the study subjects and area of residence. The chi-square test for trend was used to establish the significance of the relationships, except in the case of sport facilities per 1,000 population where p value was based on linear regression. We likewise evaluated the differences between the different characteristics of study subjects in prevalence of overweight/obesity using the chi-squared test. The association between the indicators of socioeconomic context and overweight/obesity was evaluated using odds ratios estimated by multilevel logistic regression. Given the hierarchical structure of the data presented in two levels -individuals and neighborhoods- and the possible residual correlation between persons within the neighborhoods, odds ratios were estimated using multilevel logit models which included a random effect of the intersection of origin for each neighborhood. The models were fitted using the GLIMMIX macro procedure in SAS [26]. Finally, because physical inactivity is a risk factor for overweight and obesity, a multilevel logistic regression model was used to explore whether the availability of sports facilities might account for area-based differences in physical inactivity.

## Results

Table 1 shows the relationship between the characteristics of study subjects and area of residence on the one hand, and the two indicators of socioeconomic context on the other. Significant difference was observed for overweight, obesity, educational level and occupation of primary household earner, and number of sports facilities per 1000 population. Low intake of fruit and vegetables also displayed a significant difference with per capita income.

**Table 1:** **Sample size, characteristics of study subjects and sports facilities per 1,000 population according to indicators of socioeconomic environment** ^**a**^

Sample size (n), characteristics of study subjects and sports facilities per 1,000 population	Per capita income^b^					Percentage of population with university education^b^				
	Quartile 1	Quartile 2	Quartile 3	Quartile 4	p*	Quartile 1	Quartile 2	Quartile 3	Quartile 4	p*
n	195	157	187	188		182	178	177	190	
Obesity (%)	8.2	8.3	3.8	2.1	<0.01	8.3	8.4	3.4	2.1	<0.01
Overweight (%)	27.0	33.8	30.1	16.5	<0.05	29.4	34.8	23.0	19.4	<0.01
Mean age (years)	11.0	10.6	11.0	10.9	0.535	11.0	10.8	10.6	11.1	0.265
Girls (%)	49.7	44.9	38.3	48.7	0.522	45.9	45.0	43.6	47.4	0.832
Low educational level^c^ (%)	60.0	46.2	39.5	23.0	<0.001	64.2	40.7	42.6	22.5	<0.001
Manual occupation^c^ (%)	60.6	51.6	42.9	22.4	<0.001	64.2	45.9	44.3	23.8	<0.001
Physical inactivity (%)	15.4	18.5	10.6	11.9	0.075	16.2	15.6	11.2	12.2	0.138
Low intake of vegetables/fruits (%)	44.3	46.2	34.4	34.8	<0.05	45.8	41.3	33.1	38.6	0.073
No complete breakfast (%)	86.8	85.4	91.5	84.1	0.839	87.3	88.3	87.7	84.9	0.469
Sports facilities/1000 pop.	1.6	1.1	1.6	3.0	<0.001	1.6	1.4	1.4	2.9	<0.001

^a^Based on per capita income and percentage of population with university education.

^b^The categories for per capita income are quartile 1 (<9,724.29 €), quartile 2 (9,724.29-11,149.60 €), quartile 3 (11,149.61-14,548.76), quartile 4 ( >14,548.76€). The categories for percentage with university education are quartile 1 (<14.64 %), quartile 2 (14.64-20.63), quartile 3 (20.64-35.15), quartile 4 (>35.15).

^c^Refers to educational level and occupation of primary household earner.

*The p value for subject characteristics is based on the chi-square test for trend, and the p value for sports facilities per 1,0000 population is based on linear regression.

Table 2 shows the prevalence of overweight and obesity according personal characteristics and risk behaviors. For prevalence of obesity significant differences were found according age and intake of fruit and vegetables. Children 6 to 10 years of age showed higher prevalence of obesity than children 11 to 15 years. For prevalence of overweight significant differences were found according age and sex. In the case of prevalence of obesity according intake of fruit and vegetables the magnitude of the prevalence was higher in those showing higher intake (7.0%) than in those showing lower intake (3.2%).

**Table 2:** **Frequency of obesity and overweight, in percentages, by age, sex, indicators of family socioeconomic characteristics and obesity risk behaviours**

Characteristics	Sample size (n)	Obesity	Overweight
**Age**			
6-10	324	8.6	33.3
11-15	403	3.0	21.1
p value		<0.01	<0.001
**Sex**			
Boy	396	6.1	30.8
Girl	331	4.8	21.5
p value		0.470	<0.01
**Educational level** ^**a**^			
High	416	4.1	24.3
Low	392	7.3	29.8
p value	0.062	0.059	
**Occupation** ^**a**^			
Non manual	391	6.1 25.8	
Manual	310	4.8	28.4
p value		0.456	0.251
**Time watching TV/day**			
2 hours or less	510	4.9	27.1
More than 2 hours	161	6.8	28.6
p value		0.344	0.389
**Physical inactivity**			
No	624	5.3	26.6
Yes	100	7.0	27.0
p value		0.487	0.510
**Fruits and vegetables**			
F or/and V 3 or more times/week	425	7.0	27.7
Less than 3 times/week	284	3.2	24.3
p value		<0.05	0.178
**Breakfast**			
Complete	95	6.3	26.3
Light or absent	632	5.4	26.6
p value		0.709	0.534

^a^Refers to educational level and occupation of primary household earner.

Table 3 shows the association between the two indicators of socioeconomic context and overweight and obesity. The odds ratios adjusted for age and sex in the different categories of the two indicators appear in the first column. The magnitude of the odds ratio adjusted for age and sex was hardly modified when adjustment was made for family socioeconomic position and indicators of diet and physical activity. In the case of obesity, the magnitude increased slightly when adjustment was made for indicators of diet and physical activity. After adjustment for all variables only the relation between per capita income and obesity was significant. Children and adolescents living in neighborhoods having lower per capita incomes or lower population percentages with university education had age- and sex adjusted odds ratios for overweight 1.84 (95% confidence interval (1.03-3.29) and 1.68 (0.95-2.94) times higher than those living in neighborhoods having higher per capita incomes and higher population percentages with university education, respectively. After adjusting for the different variables, the odds ratios fell to 1.80 (0.99-3.29) and 1.56 (0.87-2.79), respectively. In the case of obesity, the age- and sex-adjusted odds ratios in these quartiles of both indicators of socioeconomic context were 3.35 (1.06-10.60) and 3.29 (1.03-10.52), respectively, rising to 3.77 (1.12-12.70) and 3.42 (1.00-11.68) after adjustment for the remaining variables. In any case, the odds ratio was observed to have its greatest magnitude, not in the quartiles having a lower per capita income or a lower population percentage with university education, but rather in the third quartile, except in the case of the association between per capita income and obesity. Moreover, the magnitude of the odds ratio of the third quartile of per capita income did not showed significant differences with respect to that of the first quartile, which was used as reference.

**Table 3:** **Odds ratio (95% confidence interval) for overweight and obesity by indicators of socioeconomic environment**

Indicators of socioeconomic environment	Adjusted for age and sex		Adjusted for age, sex, and family socioeconomic position^a^		Adjusted for age, sex, family socioeconomic position and diet^b^		Adjusted for age, sex, family socioeconomic position, diet and physical activity^c^	
	OR	95% CI	OR	95% CI	OR	95% CI	OR	95% CI
**Overweight**								
**Per capita income**								
> €14,548.76	1.00		1.00		1.00		1.00	
€ 11,149.61 - 14,548.76	1.90	1.06 -3.40	1.84	1.02 -3.32	1.89	1.05 - 3.26	1.91	1.06 -3.44
€ 9,724.29- 11,149.60	2.48	1.36 -4.51	2.38	1.30 -4.34	2.43	1.34 - 4.41	2.42	1.32 -4.42
< € 9,724.29	1.84	1.03 -3.29	1.73	0.95 -3.13	1.80	0.99 - 3.41	1.80	0.99 -3.29
**Percentage of population with tertiary studies**								
>35.15	1.00		1.00		1.00		1.00	
20.64-35.15	1.12	0.62 -2.00	1.06	0.60 -1.86	1.03	0.59 - 1.82	1.04	0.58 -1.87
14.64-20.63	2.07	1.18 -3.62	1.97	1.10 -3.54	1.99	1.11 - 3.56	1.98	1.13 -3.46
<14.64	1.68	0.95 -2.94	1.53	0.83 -2.81	1.56	0.82 - 2.98	1.56	0.87 -2.79
**Obesity**								
**Per capita income**								
> €14,548.76	1.00		1.00		1.00		1.00	
€ 11,149.61 - 14,548.76	1.54	0.42 -5.60	1.54	0.42 -5.68	1.68	0.45 - 6.28	1.73	0.46 -6.49
€ 9,724.29- 11,149.60	2.92	0.89 -9.60	2.90	0.87 -9.68	3.16	0.93 - 10.70	3.10	0.91 -10.54
< € 9,724.29	3.35	1.06 -10.60	3.21	0.98 -10.56	3.66	1.09 12.29	3.77	1.12 -12.70
**Percentage of population with tertiary studies**								
>35.15	1.00		1.00		1.00		1.00	
20.64-35.15	1.00	0.24 -4.11	0.96	0.23 -3.99	0.90	0.21 - 3.80	0.92	0.22 -3.86
14.64-20.63	3.50	1.11 -11.05	3.44	1.07 -11.05	3.66	1.12 - 11.88	3.63	1.11 -11.87
<14.64	3.29	1.03 -10.52	3.08	0.93 -10.30	3.37	0.99 - 11.16	3.42	1.00 -11.68

^a^The variables of family socioeconomic position were educational level and social class of primary household earner.

^b^The variables of diet were consumption of fruits and vegetable and type of breakfast.

^c^The variables of physical activity were time watching TV and physical exercise.

Lastly, Table 4 shows the absence of a relationship between the number of sports facilities per 1000 population and physical inactivity. The odds ratio of greater magnitude was observed in the third quartile of sports facilities, although none of the odds ratio was significant. The age and sex adjusted odds ratio for the areas with the lowest number of sports facilities with respect to the areas with the highest number of sports facilities was 1.25 (0.62 to 2.79). The adjustment for other variables decreased the magnitude of the odds ratio.

**Table 4:** **Odds ratio (95% confidence interval) for physical inactivity by availability of sports facilities**

Sport facilities per 1000 population	Adjusted for age and sex		Adjusted for age, sex, and socioeconomic position		Adjusted for age, sex, socioeconomic position and per capita income		Adjusted for age, sex, socioeconomic position and percentage of population with university education	
	OR	95% CI	OR	95% CI	OR	95% CI	OR	95% CI
>1.98	1.00		1.00		1.00		1.00	
1.46-1.98	0.91	0.45 -1.86	0.79	0.38 -1.64	0.77	0.35 - 1.68	0.75	0.36 -1.57
1.01-1.46	1.35	0.68 -2.67	1.18	0.60 -2.34	1.10	0.54 - 2.25	1.15	0.57 -2.28
≤1.01	1.25	0.62 -2.79	1.17	0.58 -2.34	1.08	0.52 - 2.24	1.10	0.53 -2.25

## Discussion

Our findings show that children and adolescents residing in Madrid neighborhoods with worse socioeconomic indicators show higher prevalence of overweight and obesity that those residents in neighborhoods with better socioeconomic indicators. Even so, it is not always the neighborhoods with worse socioeconomic conditions that register a higher prevalence of these health problems. The greatest socioeconomic differences were observed for obesity. Overweight- and obesity-related risk behaviors measured in this study failed to explain these differences. Similarly, the availability of sports facilities was not related with engaging in physical activity.

Other studies have also shown that the socioeconomic context of area of residence is a predictor of children's and adolescents' body weight [1, 7, 8, 27], irrespective of family socioeconomic position [9]. In one study, per capita income was confirmed as being one of the socioeconomic indicators that most clearly predicted body weight [28]; and some studies have reported greater BMIs in areas having a lower educational level among their residents [29], though other authors have not observed this relationship [5]. A surprising finding of our study, however, was the higher prevalence of overweight and obesity in neighborhoods included in the intermediate-low quartile of both socioeconomic indicators. Another study also obtained similar results [30]. The reasons for this finding are unknown. Possibly, some obesogenic features of the neighborhood of residence, not measured in our study, are more frequent in the intermediate-low than in the low quartile.

In addition to adjusting for indicators of family socioeconomic position, our analyses were also adjusted for diet and physical activity because these are possible confounding factors in the relationship between socioeconomic context of the neighborhood and BMI [27]. Nevertheless, this adjustment failed to attenuate the association, due to the fact that most of the diet- and physical activity-related variables displayed no relationship with the indicators of socioeconomic context, overweight or obesity. Other authors also question this association [4, 17, 31, 32]. Thus, different studies in Europe show a similar access to grocery stores that is independent of neighborhood socioeconomic status [18, 33, 34].

Furthermore, there is the fact that our findings may possibly have been influenced by other characteristics which have not been included in this study. For instance, a neighborhood's social context can influence the performance of physical activity, by encouraging residents to do or discouraging them from doing physical exercise in their daily lives [19, 30, 35–37]. Similarly, the possibility should not be ruled out that, in neighborhoods with worse socioeconomic indicators, the social context is more precarious and institutional support system is not sufficiently structured, and that this in itself may be a limiting factor when it comes to engaging in physical exercise.

In another study previously conducted here in Spain, childhood and adolescent obesity also showed a greater association with indicators of socioeconomic context than did overweight [11]. In that earlier study, in which the units of analysis were provinces, the availability of sports facilities displayed a relationship with physical inactivity, and the performance of physical activity explained part of the association found. In our study, however, in which the units of analysis were Madrid city neighborhoods, the availability of sports facilities did not account for the association observed, since these were unrelated with the performance of physical activity. In this regard, it should be noted that, though different studies report a relationship between a neighborhood's characteristics and the physical activity of the children [38–40], other studies have not observed this association [5, 41]. Specifically, many authors state that children residing in the most depressed areas tend to do more exercise in parks and recreational areas [4].

When it comes to interpreting this study's findings, some of its strengths and limitations must be borne in mind. It is the first study to examine the proposed hypothesis in a city in a southern European country. In addition, a neighborhood-based study avoids limitations posed by the larger geographic units. For example, when states, regions or provinces are studied, the units of analysis are more homogeneous in terms of availability of facilities and residents' socioeconomic profile, and it is more difficult to detect a relationship between two variables if a relationship did exist [27]. Thirdly, based on measured weight and height, BMI was used for the purposes of this study; and BMI is considered the indicator of choice for measuring obesity in children aged 2 to 19 years. Furthermore, use was made of Cole's criteria, which allow for international comparisons between the children and adolescents of different populations [22].

As regards the possible limitations should be noted that this is a cross-sectional design which could affect the direction of the association investigated, though it is highly unlikely that obesity in children determine the neighborhood where they reside. Similarly, one should not rule out the possibility of there being unknown errors of measurement in the collection of data on risk behaviors, diet and physical inactivity; even so, there is no need to suppose that such errors would differ in terms of neighborhood of residence. In any case, the validity of instruments of measurement about diet and physical activity may not be appropriate. The measurement on diet is raw and in the measurement of physical activity has not been determined whether participants are meeting the recommendations of physical activity, because the intensity and duration of activity is unknown. This limitation may explain the absence of a gradient in these behaviors according socioeconomic context and therefore these behaviors do not contribute to explain the association investigated.

## Conclusion

In summary, the findings of this study show a relationship between a neighborhood's socioeconomic context and obesity among children and adolescents in the city of Madrid. This association is not explained by family socioeconomic position and measures used on unhealthy diet or physical inactivity; nor does the availability of sports facilities appear to play a role in this relationship.

## Data sharing

The data used in this study are available upon request to Enrique Regidor
